# Towards an understanding of community-engaged efforts in addressing the youth substance use in the Democratic Republic of the Congo

**DOI:** 10.1186/s12889-025-25809-z

**Published:** 2025-12-19

**Authors:** Minghui Deng, Fangyuan Li, Omolola A. Adeoye-Olatunde, Nan Kong, Pengyi Shi

**Affiliations:** 1 Purdue University College of Pharmacy, 640 Eskenazi Avenue, Indianapolis, IN 46202 US; 2 Purdue University School of Industrial Engineering, 315 N. Grant Street, West Lafayette, IN 47907 USA; 3 Purdue University Weldon School of Biomedical Engineering and Edwardson School of Industrial Engineering, 206 S Martin Jischke Dr, West Lafayette, IN 47907 USA; 4 Purdue University Daniel School of Business, 403 W State St, West Lafayette, IN 47907 USA

**Keywords:** Youth substance use, Mixed methods design, Community-Engaged research, Democratic Republic of the Congo, Integrated conceptual framework

## Abstract

Youth substance use is a growing concern in the northeastern Democratic Republic of the Congo (DRC). This study aims to enhance understanding of community training programs that prevent youth substance use by designing a comprehensive process map that illustrates the roles and interactions of key entities and individuals, including local churches, community workers, and nongovernmental organizations (NGOs). We also conducted surveys and interviews to further explore community-led programs. Five training centers have been built to help young adults gain professional skills and prevent them using substances. Our findings identified the four key groups in this process: youths, leadership team, teachers, and youth leaders. This study emphasizes the critical role of community engagement in addressing youth substance use and highlights the importance of further research opportunities for using technology to enhance training centers’ operational efficiency and impact. By addressing identified challenges, these training centers can become more effective in empowering youth and preventing substance use in the region.

**Supplementary Information:**

The online version contains supplementary material available at 10.1186/s12889-025-25809-z.

## Highlights

Youth substance use drives social issues like poverty and school dropout in Aru, DRC. Community training programs provide early intervention through education and skills training to prevent young adults from turning to substance.

Process mapping was implemented to thoroughly analyze and document the workflows and activities of the community-based substance use prevention and rehabilitation programs.

This study utilized a mixed-methods design, integrating open-ended surveys and interviews for qualitative insights, followed by closed-ended surveys for quantitative analysis.

**Supplementary Information:**

The online version contains supplementary material available at 10.1186/s12889-025-25809-z.

## Introduction

### Youth substance use in the Democratic Republic of the Congo (DRC)

**Fig. 1:**
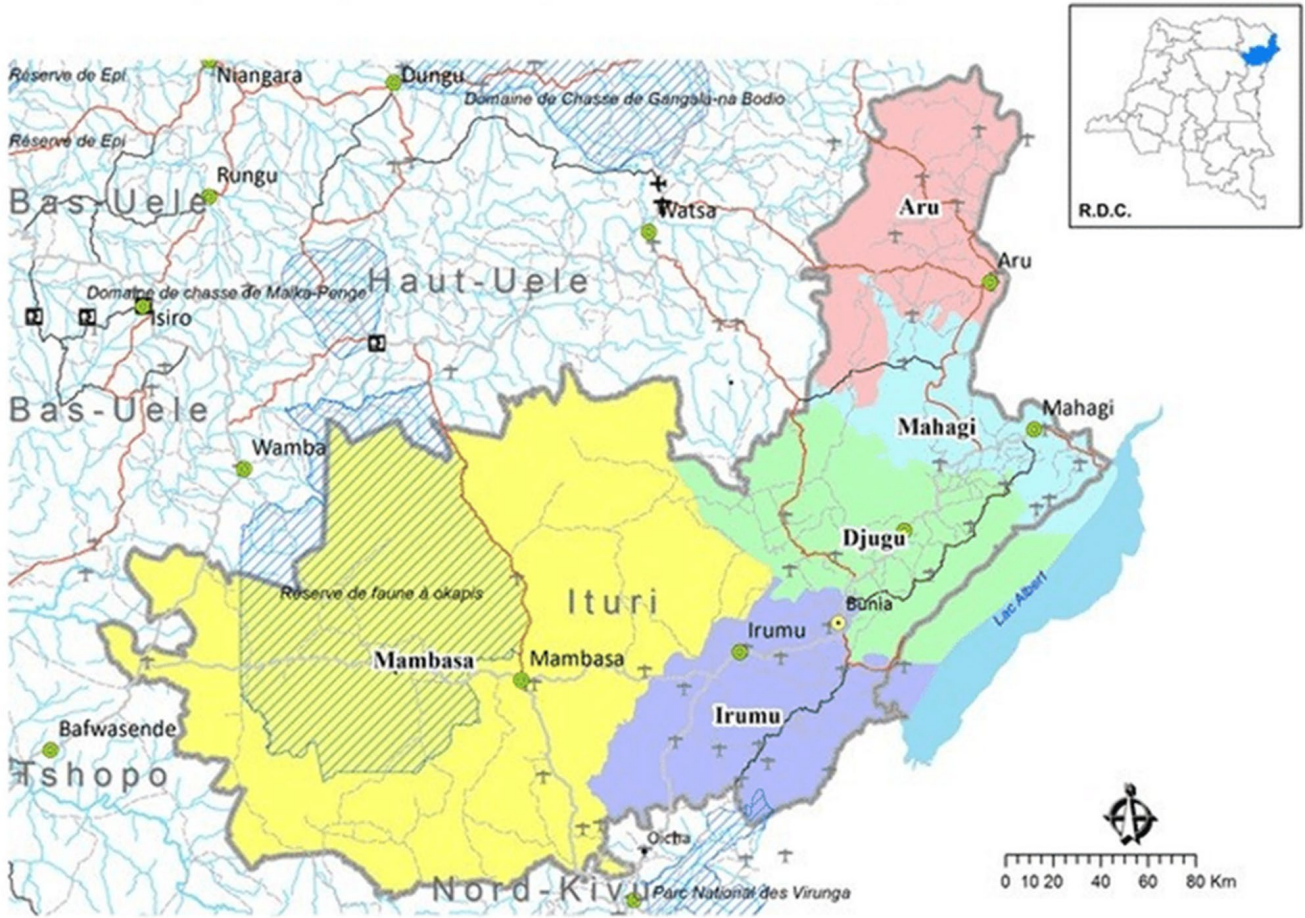
Map of Ituri Province. Source: Cellule d’Analyse des Indicateurs de Développement [4]

Youth substance use has been both a result and a cause of complex social problems such as poverty, school dropout, militant groups, and lack of educational and economic opportunities in the Democratic Republic of the Congo (DRC) [1–3]. Aru Diocese, DRC, is a region covering three agglomerations in the northeast corner of Ituri province and four neighboring provinces. Fig. 1 shows the map of Ituri Province [4]. This area faces an increasing burden of youth substance use [2, 3]. The habit of consuming illicit drugs and alcohol in high concentrations was introduced to the Aru Diocese by armed groups during the inter-ethnic and tribal wars in 2003 [5]. It was expanded through the village trading centers, night-time activities, and rampant smuggling by traffickers later, and now has led to youth substance use in the larger area along the border. Drugs and substances are available locally on farms or from neighboring Uganda and South Sudan. The easy access to drugs and substances worsens the situation of youth substance use. Youth substance uses harms the communities by increasing healthcare costs, especially for low-income countries like DRC, which already lack medical resources. It also induces and aggravates other social problems, including high crime rates [6], risky sexual behaviors [7], and strained relationships between teenagers and adults [8].

In this study, the term “youth” follows the World Health Organization (WHO) definition of individuals aged 15–24 years. The original research question and study design were conceptualized to understand substance use among youth (15–24 years) in community training centers. However, during data collection, we found that all participants were aged 18 years and older. While the study was designed with a broader “youth” focus, the findings specifically represent young adults (ages 18 and above) as defined within this local context. Importantly, the local authorities and training centers in the Aru Diocese consistently classify all enrolled individuals as youth, regardless of age, and our terminology reflects this community definition.

### Community-engaged efforts to address youth substance use in the DRC

Since 2005, the Diocese of Aru has been actively advocating for the advancement and consolidation of social justice, education for responsible citizenship, and education for peace. The diocese has implemented a series of programs aimed at providing youth with essential skills in literacy, money management, and conflict resolution, thereby engaging them in community development. [4] The DRC is heavily influenced by the Christian religion, which is practiced by almost 96% of the people in the country. Within this context, religious networks have emerged as important elements of civil society and as powerful forces for social mobilization [9]. Collaborating closely with local churches, community leaders, trained staff, community workers, and non-governmental organizations (NGOs), these community-based initiatives offer services and support to youth at-risk for substance use through training programs. Current community training programs provide early intervention, including basic education and skills training to prevent young adults from turning to substances. Additionally, they offer detox and recovery services for those who are currently struggling with substance use. The preventive role of the training centers is informed by local community partners. Based on community perspectives, the training centers function as protective community structures that may reduce risk factors for substance use by providing education, vocational training, and social support in a stable and constructive environment. These programs have achieved significant success in targeted areas, involving a total of 1765 individuals aged 20 to 50, to acquire literacy and numeracy skills [2]. In light of these achievements, there is a critical need for a systematic understanding of (i) how the community training programs address the challenges of youth substance use and (ii) barriers to program expansion and trainee retention and how they might impair performance, sustainability, and advancement of community program efforts toward achieving greater social benefits. Therefore, researchers from Salama University in Aru, DRC, collaborated with Purdue University in the United States to address these real-world gaps. The connection between the community team in Aru and researchers at Purdue University was established through the Purdue Shah Family Innovation Lab in the Purdue Global Engineering program. The objectives of this study were to (i) develop a comprehensive process map outlining how different key individuals and organizations interact with each other during the process of youth substance use prevention in the DRC, and (ii) identify current bottlenecks (barriers) and needs to help expand the training center and retention of students. The study was the initial stage of a larger effort to design a digital solution. The primary purpose of this study is to present a community-engaged, mixed-methods process-improvement study that documents the development and implementation of community-based youth substance use prevention initiatives. Rather than evaluating the effectiveness of these programs, this study focuses on examining the implementation process, stakeholder engagement, and contextual barriers that shape how these initiatives operate within the community.

## Methods

### Study design

This cross-sectional observational study applied an exploratory sequential mixed methods design. The mixed methods design begins with qualitative data collection, followed by quantitative data collection [10]. Open-end surveys and semi-structured interviews for the qualitative component and close-end surveys for the quantitative component were used for data collection (Appendices A to F). Qualitative open-ended surveys and semi-structured interviews were used first to gather in-depth data, allowing participants to express their thoughts, attitudes, behaviors, and experiences in their own words. These qualitative studies were also used to facilitate a better understanding of the context with a smaller sample size and limited language resources.

The study originated from the Purdue Shah Family Innovation Lab’s collaboration with the Aru leadership team, who identified youth substance use as a local priority and advised on context-appropriate research methods. This collaboration guided the selection of methods feasible for implementation in a low-resource setting while ensuring cultural and linguistic relevance. The issue of youth substance use in low-income country border war zones is a persistent but under-investigated challenge. Qualitative approaches were most appropriate to start with, as researchers lacked sufficient contextual information and empirical qualitative research findings could better inform the design of relevant closed-end survey questions to address study objectives. ^11–14^ A mixed methods consolidation approach was then used to synthesize qualitative and quantitative findings, which subsequently informed process mapping. Process mapping was used to systematically analyze and document the workflows and activities involved in the community-based substance use prevention and rehabilitation programs in Aru, DRC.

The study team at Purdue University recognize that conducting research in a post-conflict, low-resource setting raises additional ethical considerations beyond formal approval. Therefore, this research was conducted through a community-engaged partnership with the Aru leadership team, Salama University, and local community workers who were integral to the study’s implementation. These collaborations promoted transparency, local ownership, and mutual respect throughout all stages of the project. This study was approved by the ethics committee of Purdue University (IRB: 2021 − 1027). The reporting of this study followed the Strengthening the Reporting of Observational Studies in Epidemiology (STROBE) guidelines by Cuschieri (2019) and Good Reporting of a Mixed Methods Study (GRAMMS) [15, 16].

### Community-engaged research approach

The study used a community-engaged research approach. Community-engaged research involves active collaboration between researchers, key entities, and community members in several aspects of the research process. [17] The key entities and community members engaged in this study consisted of the US and Aru research teams, local Aru religious leaders, community workers, teachers, students, youth leaders and the NGOs working with local communities. Community-engaged research was used to (1) gain familiarity with key individuals, organizations, and their relationships, (2) understand their workflows in addressing youth substance abuse and identify the facilitators and barriers for the support team, and (3) link the observations with principles of designing interventions for complex social problems [18].

Community partners played an active and collaborative role throughout the research process. The project was initiated by the Aru leadership team. The U.S. based research team met biweekly with local partners during the planning phase to co-develop the research questions. The study’s initial focus on a treatment program was revised to emphasize education and prevention, reflecting local priorities and feasibility. Community partner’s input ensured that the study aligned with locally recognized needs, available infrastructure, and community readiness. Community partners also contributed to the development and refinement of survey questions. Although the first open-ended survey was created by the U.S. team, feedback from community partners led to revisions to improve accessibility and literacy compatibility. Local collaborators reviewed all questions for language appropriateness, ensuring that questions reflected local terminology. Community partners also translated surveys into regional languages and managed printing, distribution, and collection from teachers, students, and youth leaders. They also transcribed and translated responses into English for further analysis. Regular communication between the Purdue and Aru teams via weekly emails and WhatsApp facilitated ongoing feedback. These communications allow community partners to clarify findings and provide contextual interpretation. Finally, community partners helped present study results to local groups to support knowledge sharing and ensure that the research findings were returned to the community.

### Study setting and participants

The study took place in Aru, Democratic Republic of Congo, with specific attention to the Totonga training center and Elikiah training center located at Aru City. Data collection took place from November 2022 to November 2023. Participants were selected based on specific criteria. The inclusion criteria included community workers, students, teachers, and youth leaders actively engaged in local training centers within the Diocese of Aru, DRC. Participants were invited to voluntarily participate in the survey and/or interviews and required to provide informed consent to participate in the study. Additionally, participants needed to understand and respond to the survey and interview questions in the language used for data collection, with translated versions available in French and Lingala. The exclusion criteria consisted of individuals who did not meet the inclusion criteria. Initial participants were identified through the Purdue Shah Family Innovation Lab collaboration and included individuals from the Aru leadership team. These individuals provided initial insights and helped identify further participants. Subsequent recruitment involved teachers, students and youth leaders from local training centers, with assistance from the Salama University team and local community workers.

The study was designed as an open community-based approach in which all individuals enrolled in participating training centers were invited to participate. No explicit age-based inclusion or exclusion criteria were applied. The study was originally designed to explore substance use among youth aged 15–24 years, consistent with the World Health Organization (WHO) definition of youth. However, during data collection, we found out that all enrolled participants were aged 18 years and older. The research team continue to use the term “youth” throughout the study to align with the local classification used by community partners, who consider all training center enrollees as “youth” regardless of their specific age.

### Integrated conceptual framework

**Fig. 2:**
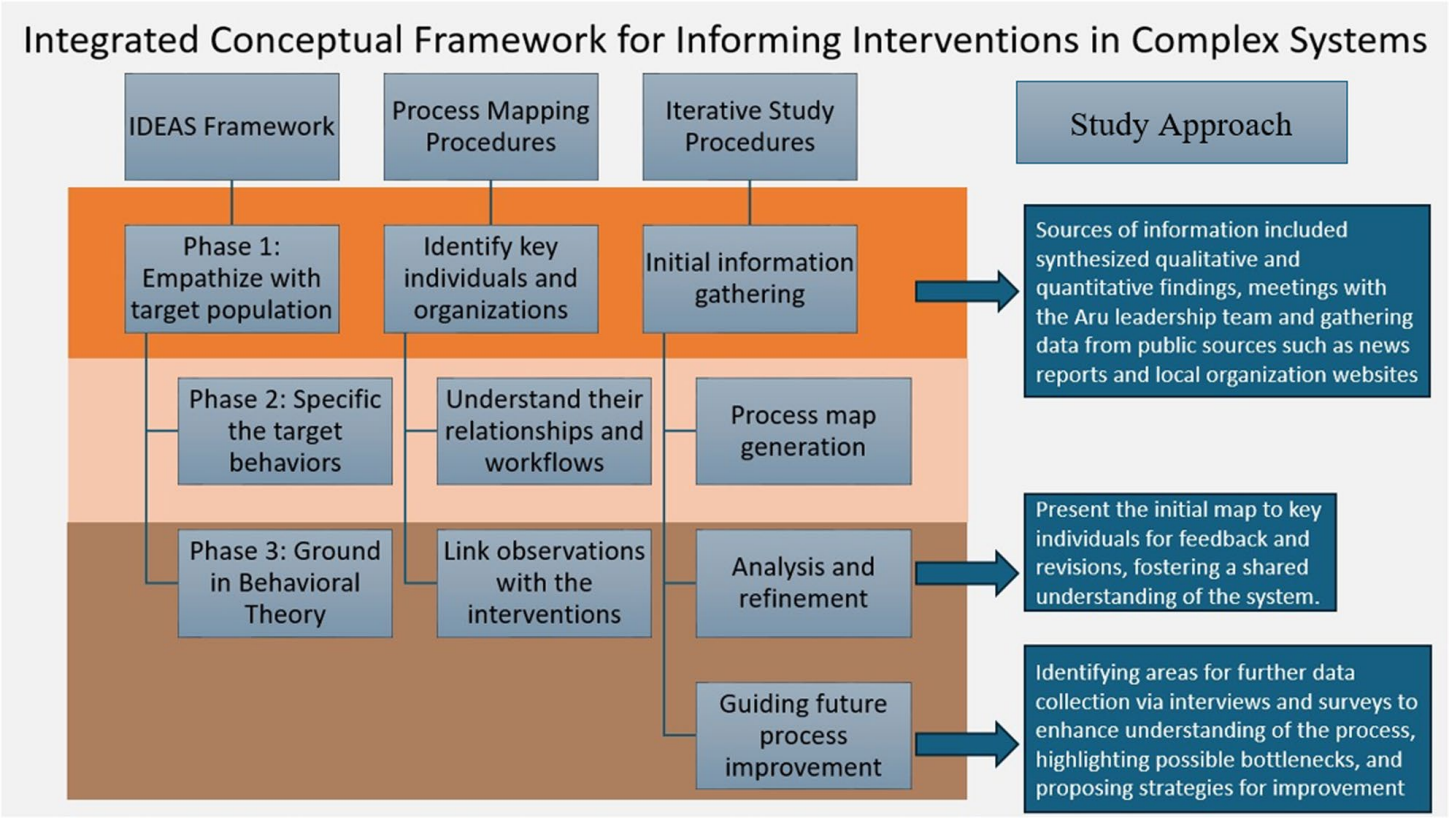
Integrated conceptual framework for informing interventions in multifaceted systems. The figure includes how the framework was operationalized for this specific study approach as an example. Abreviations: IDEAS (Integrate, Design, Assess, and Share)

The study was guided by our Integrated Conceptual Framework for Informing Interventions in Multifaceted Systems (Fig. 2). It combines elements from:

1. Integrate, Design, Assess, and Share (IDEAS) framework created [18].
2. Continuous Quality Improvement (CQI) toolkit – Process Mapping [19].

*First*,* the IDEAS framework served as the conceptual foundation for the study design*. It offers an in-depth understanding of multifaceted systems by identifying key individuals, sequences of actions, decision points, and information flows [19]. This framework is one of the most comprehensive guidelines for developing and evaluating digital interventions. It comprises ten phases grounded in four overarching stages (Integrate, Design, Assess, and Share). We designed the study to fulfill three phases of the first stage “Integrate”:

- Phase (1) empathize with the population of interest.
- Phase (2) specify the target behaviors.
- Phase (3) ground in behavioral theory to integrate insights from users and theory.

*Second*,* process mapping functioned as the procedural tool for operationalizing these conceptual insights within the local system.* It offers an in-depth understanding of multifaceted systems by identifying key individuals, sequences of actions, decision points, and information flows [19]. Following the framework by [20], the process map development involved four major steps:

(I) Information gathering and initial process identification; source of information included synthesized qualitative and quantitative findings, meetings with the Aru leadership team and gathering data from public sources such as news, reports and local organization websites.
(II) Process map generation, using the gathered information.
(III) Analysis and refinement: present the initial map to key personnel for feedback and revisions, fostering a shared understanding of the system [21].
(IV) Guiding future process improvement: identifying areas for further data collection via interviews and surveys to enhance understanding of the process, highlighting possible bottlenecks (areas for improvement), and proposing strategies for improvement.

Given the multifaceted and under-researched nature of our study setting, process mapping was crucial in our iterative framework. This approach provided a detailed understanding of the operational context, which informed the development of surveys and interviews to reflect on-ground realities and operational dynamics, ultimately helping to finalize the process map. Fig. 2 provides an overview of the integrated conceptual framework aligned with the operational study procedures.

These frameworks were selected by the U.S. based research team. The U.S. based team comprised three researchers with engineering backgrounds in healthcare operations and service systems design, and two researchers with pharmacy and public health backgrounds in implementation science and community-engaged research. These disciplinary perspectives contributed methodological rigor and a systems-oriented view. The three researchers with engineering backgrounds provided guidance on process mapping and systems analysis, and the two researchers with community-engaged research expertise contributed to the mixed-methods design, framework selection, and data analysis. The combination of these perspectives allowed for a structured, process-oriented approach grounded in community-engaged principles. Framework selection occurred during the study’s planning phase and was not formally discussed or validated by community stakeholders in Aru. However, both frameworks were adapted in practice through the study’s iterative feedback and communication process with community partners.

### Data collection

As data collection was an iterative process, some of the formative results are presented here to contextualize process and rationale for iterative data collection procedures.

#### Open-ended surveys

We started our research by designing open-ended surveys to be administered via paper to community workers. We gathered comprehensive information through multiple channels to form a basic understanding of the study area. Those channels included online Google searches and conversations with Aru collaborators working in the Democratic Republic of Congo. This step helped the research team be more aware of the local language, religious beliefs, transportation, and cultures. After the basic information gathering, we created an open-ended survey that is informed by the Integrated Conceptual Framework and designed to gain a deeper understanding of the target population and their needs. The survey consisted of questions spanning five domains (1) Key individuals and organizations, (2) background information gathering, (3) perceived challenges, (4) roles and workflow, and (5) comments on the proposed process mapping. Contextual descriptions for questions were provided to ensure that the respondents understood the intention behind each question and to get a basic understanding of the situation in Aru while limiting biases.

Two members on the Aru leadership team identified by the Purdue Shah Family Innovation Lab connection were invited to participate in the open-ended survey study by submitting typed responses in WORD format (Appendix A). No monetary incentives were offered. Through the initial round of surveys with the leadership team, we gained insights into the process, clarified the roles of each key individual, and obtained comments on the initial process map. Furthermore, we identified teachers in the training centers as key individuals and designed another round of an open-ended surveys (Appendix B) to gather more contextual information from teachers in the training centers.

With the help of the Salama University team, we connected with more key individuals and collected information. We conducted our first round of open-ended surveys with the recruited teachers in the training centers to gather more insights. Participants were recruited with the help of local community workers and Salama University researchers. We started with six teachers from the two training centers in the urban area for the open-ended survey. The survey included questions about teachers’ daily activities and current barriers (survey details are attached in the Appendix B) This open-ended survey was sent to the local team (community workers) via email in English in WORD format. Translation of surveys was conducted by local community workers proficient in English, French, and the local languages. To maintain linguistic and contextual accuracy, the translators and the Purdue research team jointly reviewed and discussed any discrepancies until consensus was reached on meaning equivalence. After translating the survey, they then distribute it to the teachers in paper format. Teachers completed the surveys by writing on printed copies. The local community workers collected the responses, translated them (and transferred them back to WORD documents if needed), and sent them back to the research team at Purdue University. Each survey took approximately 25–40 min to complete, and no incentives were offered to participants.

#### Feedback and semi-structured interviews

We provided weekly updates to the two community leaders via email and WhatsApp messaging. Through these communications, we found some inconsistencies between the feedback from community leaders and the open-ended survey responses, specifically regarding the barriers to expanding training centers, that is, whether there was insufficient student demand or insufficient teaching capacity. To gain a more accurate understanding, we arranged a semi-structured individual interview with one of the survey participants who volunteered. We consented and scheduled a one-to-one Zoom interview with the teacher who agreed to participate. (Past meetings with the local team had been conducted via Zoom, so the feasibility of a Zoom interview had been tested.) A professional interpreter joined the interview to help translate the conversation. During the Zoom interview, we turned on the real-time closed caption function after getting consent from the participants and saved the captions as transcripts for further analysis. After the interview, we provided copies of transcripts to the research participants and invited them to comment on or correct any misinterpretations. Through this interview we confirmed that there was sufficient student demand, while the teaching capacity and transportation to training centers were main issues.

Then we moved on to Phase 2 of the IDEAS framework, which involved specifying the target behavior with a focus on understanding more details about the challenges. The results obtained from the interview proved to be very helpful in crafting questions for subsequent rounds of surveys. For example, one of the responses received from teachers was that their students were facing transportation challenges. We then narrowed down this issue to more specific queries, such as, “What is the average commuting time for students to come to the training center?”

#### Feedback and closed-ended surveys

After conducting the interviews, we requested feedback from the community partners via email. Based on their input, we converted the second round of surveys from open-ended paper-based surveys to closed-ended surveys with multiple-choice questions. Feedback from community partners has led to key revisions to our data collection tools, such as simplifying question wording, adding culturally relevant response options, and transitioning from open-ended to closed-ended formats to accommodate local literacy levels. The results from the open-ended surveys were not satisfactory, largely due to the language barrier. Therefore, with the support of the community partners, we decided to make this change to obtain more precise data. The responses from these surveys were systematically compared and analyzed to generate a comprehensive and cohesive assessment of the youth substance use issues in the Aru region. The second-round surveys focused on the daily workflows, barriers encountered, and suggestions for program improvement from each group, forming the basis for a thorough analysis. We created three types of surveys for youth leaders, teachers, and students. The surveys were emailed to the local team (community workers) in WORD format. Local community workers helped translate the surveys and distribute them to everyone in paper format. Participants were chosen by local community workers based on volunteering. All participants completed the surveys by handwriting on printed copies. The local community workers helped collect the responses, translate them, and send them back to the research team at Purdue University. Each survey was expected to take approximately 20 min, and participants were not offered incentives. In the later rounds, we chose to use all close-ended questions because we were surveying more students and youth leaders, who often have lower literacy levels in reading and writing compared to the teachers surveyed earlier.

All data in this study are self-reported by participants through either surveys or interviews. The U.S. based research team did not conduct any direct field observations. Instead, all participant recruitment and data collection activities were coordinated and implemented by local community workers. The Purdue research team relied on local community workers for on-site facilitation and participant engagement while they provided oversight in study design, instrument development, and analytical interpretation to ensure methodological rigor.

### Data analysis

**Fig. 3:**
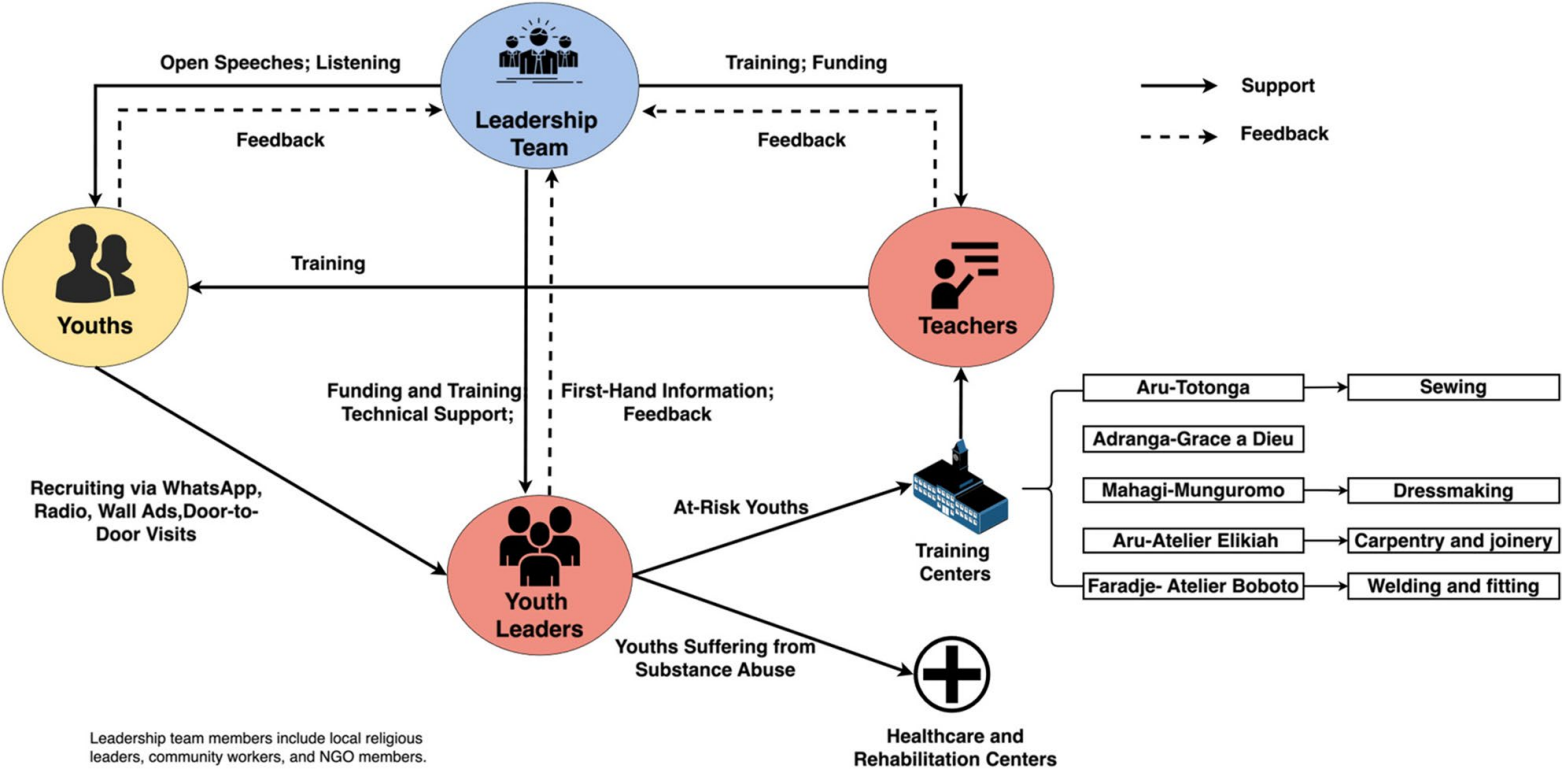
Process map of the system

**Fig. 4:**
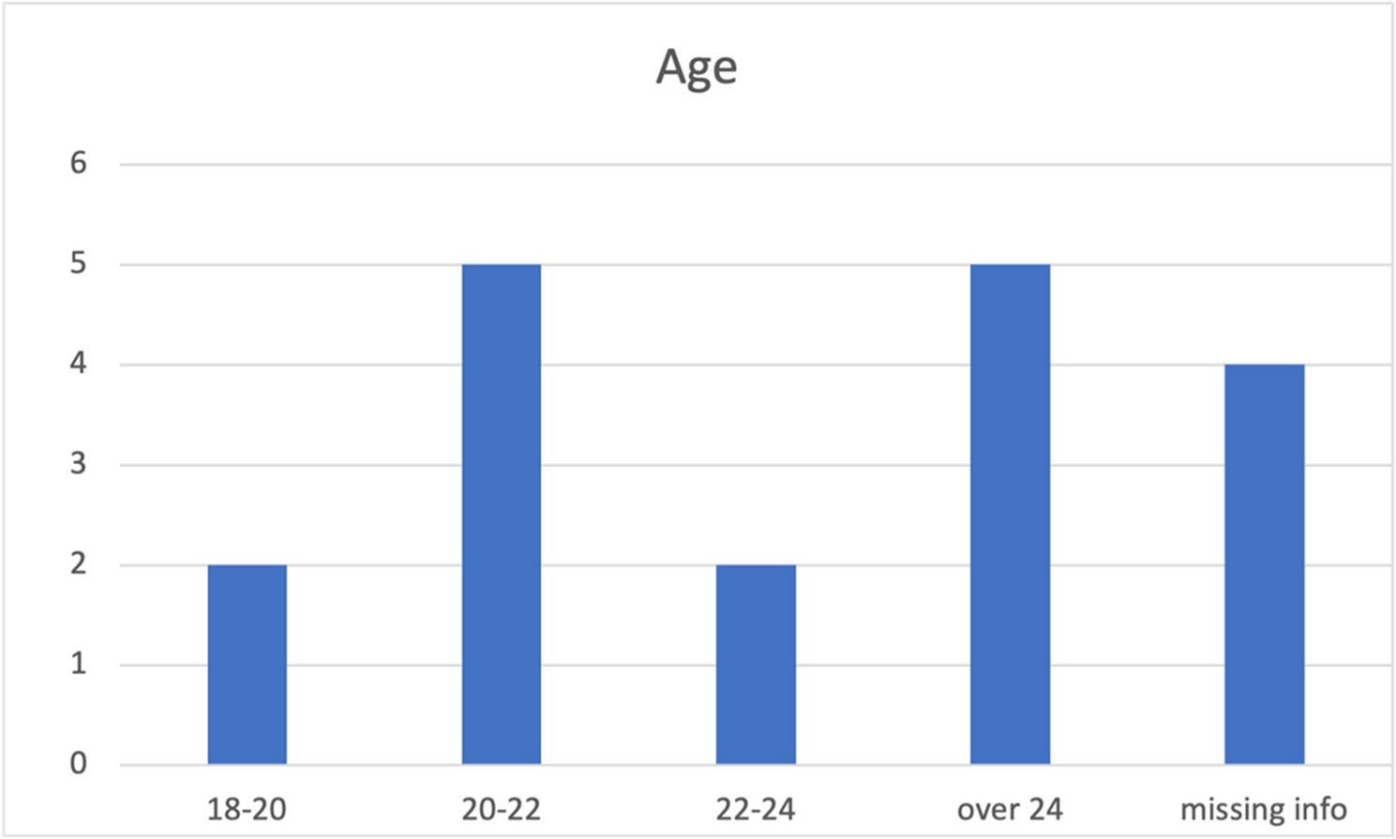
Distribution of student ages

**Fig. 5:**
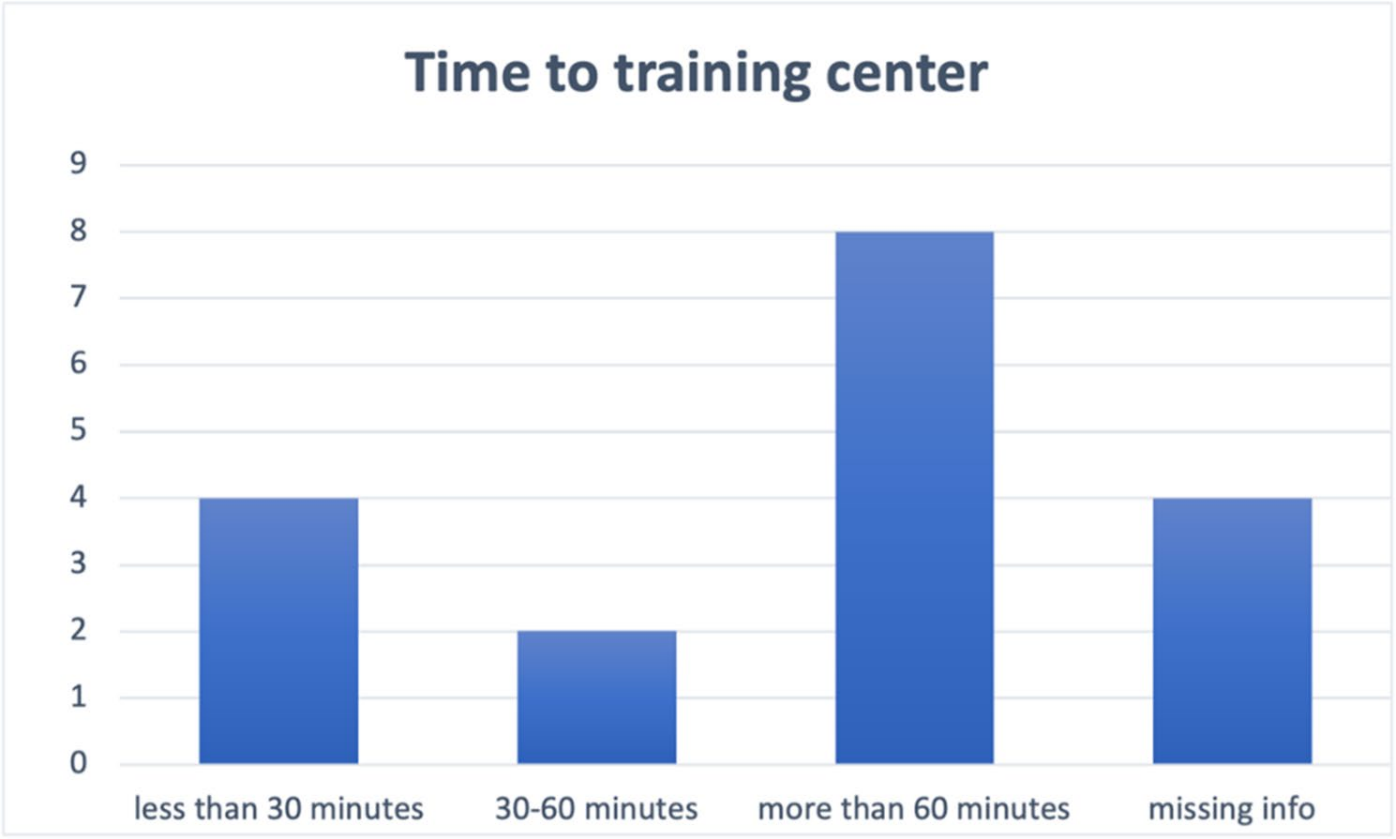
Distribution of commuting time to the training center

**Fig. 6:**
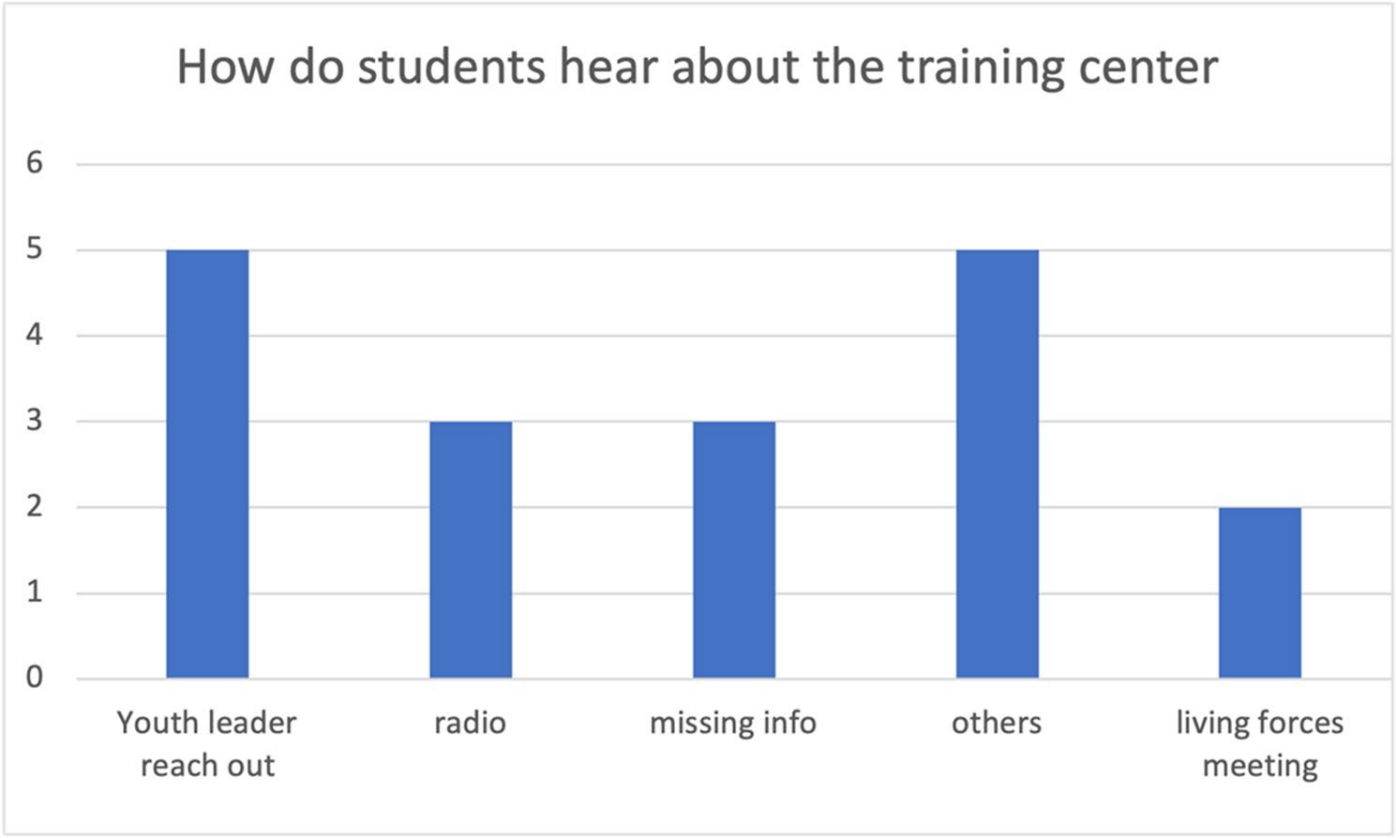
Sources of student awareness about the training center

**Fig. 7:**
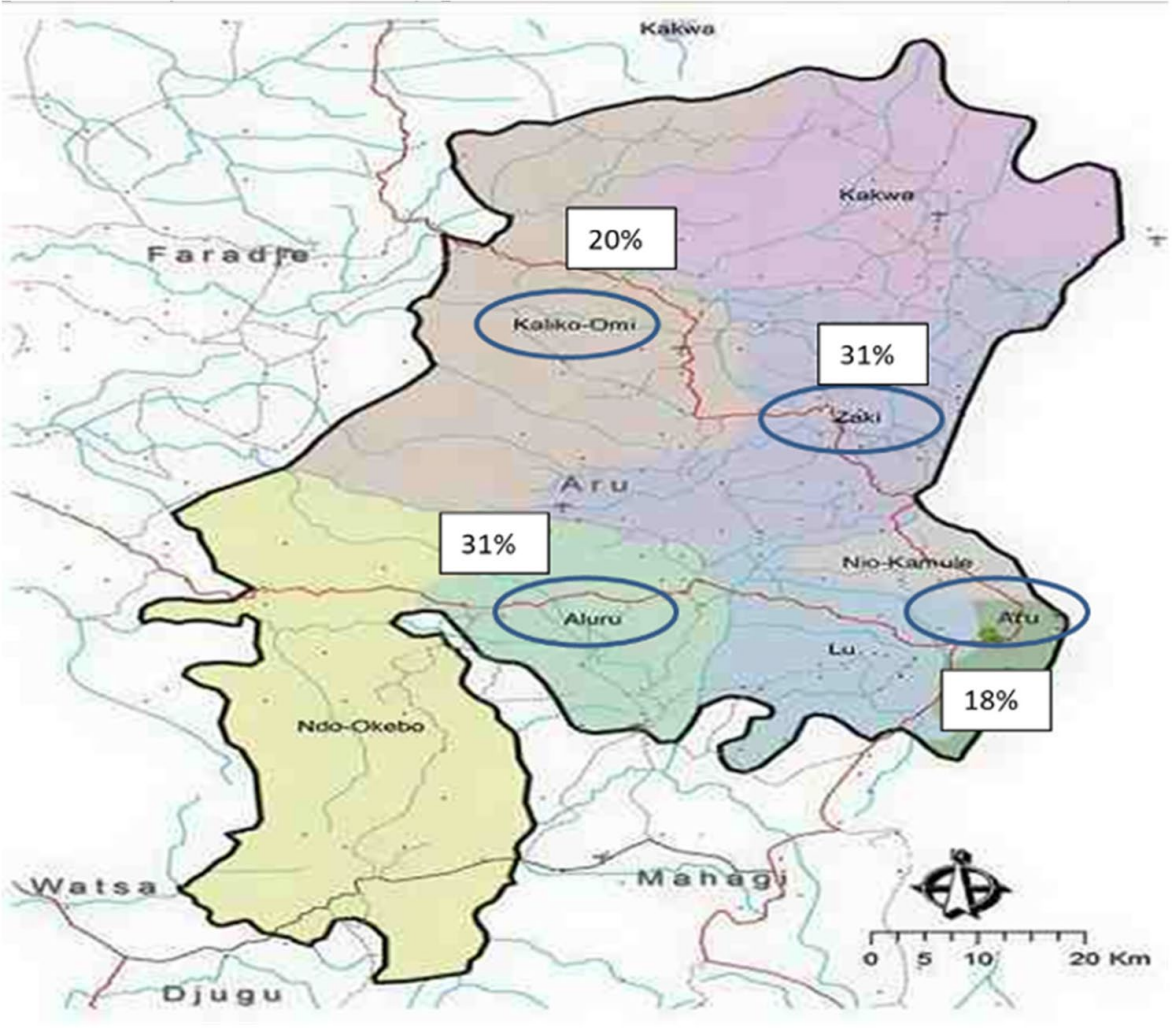
Geographic distribution of youth leaders in the Aru Territory (We acknowledge the limited image quality of the map. Despite extensive efforts, higher-resolution maps with accurate chiefdom boundaries within the Aru Territory were not publicly available, and this version provided by our community partner remains the most reliable source.)

In our study, we used a comprehensive approach to analyze the data. Qualitative data analysis utilized inductive approaches. Inductive approach involves reviewing the data to identify emerging codes, categories, patterns, and themes. [22, 23] In this study, we analyzed the open-ended survey responses, recorded keywords from each response in Word documents, and summarized them in a table. We then use the summarized data to create the process map (Fig. 3) and to guide our close end questions. Quantitative analysis for closed-end survey results consisted of counting the number of respondents for each answer and created corresponding graphs in Excel (Figs. 4, 5, 6 and 7). For the interview results, interview transcripts and video recordings were carefully reviewed to ensure accuracy and completeness. The researcher who conducted the interviews reviewed and compared each transcript against the corresponding video recording to verify the accuracy of the content and contextual details. A graduate student assisted with coding. The analysis followed an inductive thematic approach, moving beyond keyword identification to identify recurring concepts, patterns, and relationships within participant responses. Both researchers independently reviewed the transcripts and discussed emerging themes to ensure consistency and reliability in interpretation. After the preliminary coding, the coded transcripts and summaries were shared with the interviewees and the translator for verification and feedback, allowing participants to confirm the accuracy of their statements and the contextual meaning of their responses.

## Results

### Process map

Based on findings described in detail below, the process map in Fig. 3 illustrates observed systematic interactions among key individuals and the pathways of youths within the system. The upper left side of the map shows key individuals and their interactions, while the lower right side displays the pathways of youths within the system and facility information. Circles identify four types of key individuals involved. They are “Youths,” “Leadership Team,” “Teachers,” and “Youth Leaders.” There are five training centers in the study area. The five centers are named after their locations. Aru-Totonga Training Center teaches youths sewing, Mahagi-Munguromo Training Center teaches dressmaking, Aru-Atelier Elikiah Training Center teaches carpentry and joinery, and Faradje-Atelier Bototo Training Center teaches welding and fitting. We are unsure about the skills taught at Adranga-Grace a Dieu Training Center. Solid arrows represent the flow of youths, or the support provided by the stakeholder at the head of each arrow to the stakeholder at the tail of the arrow. Dashed arrows represent feedback among key individuals. Youths include those already suffering from substance abuse and those at risk. The leadership team consists of local religious leaders, community workers, and NGO members, playing a crucial role in coordinating and guiding the actions of other key individuals. The leadership team provides funding and training opportunities to youth leaders and teachers in the training centers. They also give open speeches to inspire youths and listen to the voices of youths. In the Aru territory, religion is an essential part of life, and religious organizations typically work with the government to promote the socio-economic and spiritual well-being of the community. The Diocesan Development Office Department (BDD), operating under the Social Welfare Service (SBES), is directed by a Diocesan department coordinator who works with a team of 36 contracted community workers. Non-governmental organizations, such as World Concern and Five Talents UK, also assist local communities in identifying, developing, and expanding local resources and creating new businesses and opportunities.

Youth leaders and teachers are practitioners at the center of the process. Youth leaders are volunteers affiliated with churches or other volunteer groups, such as the Local Peace Committee. They connect with local communities and recruit youths in markets and churches through WhatsApp messaging, wall advertisement, broadcasting via local RCE (Radio Communautaire Etoile), and even door-to-door activities. They also reach out to local associations, including training centers, to discuss the possibility of placing youths at those associations. Once youths are recruited, youth leaders conduct screenings and send youths suffering from substance abuse to healthcare and rehabilitation centers, while at-risk youths are sent to training centers. Teachers in the training centers are locals with expertise, and they teach youths not only livelihood skills but also problem-solving techniques, such as reading, writing, and negotiating with others. Youth leaders and teachers provide the leadership team with first-hand information and feedback based on their interactions with youths. This feedback helps the leadership team make better-informed funding decisions.

### First round open-ended surveys and semi-structure interview

Two responses were obtained from our initial round of open-ended surveys with *community workers*, one from a religious leader and the other from a staff member at an NGO working with local communities. Based on those responses, we were able to enrich the process map by adding (1) a new stakeholder – Administrative Authorities (Political authorities and Customary authorities), which are responsible for enforcing laws on drug use, making rules, proving security, etc.; (2) detailed descriptions of key individuals and their roles, e.g. teachers at the training centers include local residents and individuals with specific skills who live closer to the border, and they train and enlighten youths, analyze and improve the process, as well as supervise any noncompliance; (3) background information, e.g., the number of youths affected by substances abuse, factors leading to youth substance abuse, information about the infrastructure, etc.; and (4) identified challenges, e.g., lack of training centers, no means of transportation, finances, technology or materials, no documentation on drug addicts, etc.

Six responses were obtained from *teachers* at two of the five training centers. Four teachers are associated with the Aru Totonga center, while the other two are associated with the Aru Elikiah center. Teachers at the Aru Totonga Training Center are predominantly female, aged between 29 and 40. They teach students to sew clothes and sell them to residents nearby for supplemental income. The students usually stay at this training center for about six months to learn sewing skills. Teachers at the Aru Elikiah Training Center are mainly male, aged between 54 and 71. They teach students about carpentry and the students usually stay there for six to ten months.

We noticed some inconsistencies in the responses and found that some answers were too brief, possibly due to mistranslation or unclear questioning. As a result, we scheduled a Zoom interview with one of the teachers to gain a clearer understanding of the local situation.

A teacher from the Aru Totonga Training Center participated in our semi-structured interview via Zoom. This 40-year-old male interviewee teaches sewing at the center and has a class of 33 students. Before joining this center, he taught at another school and underwent special training to teach here. Although he focuses solely on sewing, the center offers various other subjects. The Aru Totonga Training Center has four other teachers handling in different classes, including a class where students study the Bible. Each class runs for two hours, with practice time available between and after each class period. Students may also stay after school to practice their sewing skills. In addition to teaching, teachers at the center earn their income by selling clothes to locals. Current challenges include limited space in the classroom despite a high student population and a lack of sewing machines, scissors, and books. Students need to share these resources with others during the class. The interview lasted approximately 40 min, but our call was prematurely disconnected due to unstable internet connectivity from the Aru side.

### Second-round closed-ended surveys

In the second round of surveys, our participants included *youth leaders*,*teachers*,and*students*. Due to the geographical distance between the U.S. based study team and the sites in Aru, DRC, surveys were distributed by local community partners (local community workers and Salama University researchers). Because there is no centralized registry of enrolled students and youth leaders and transportation barriers limited access to certain areas, we were unable to determine the total number of individuals invited. Consequently, response rates could not be calculated for these two participant groups. We were able to confirm that all six teachers at the participating centers completed the survey, resulting in a 100% response rate for the teacher population. The six responses from *teachers* located in two training centers, like the first round. Regarding their daily schedule, both training centers hold classes five days a week, starting at 8 am. The teachers emphasized several challenges they faced at that time, such as transportation issues and the insufficiency of facilities and equipment to accommodate additional students. Since students are responsible for commuting to the training centers by themselves, students who live far away from the training centers have difficulties coming to class five days a week, and the rainy season further poses a significant obstacle to their attendance. Additionally, they identified other barriers, including a lack of teaching materials, electricity, and space within the training centers. They observed an increasing demand among students enrolled in the classes. However, the limited capacity of these facilities prevents students from learning new skills. To address this challenge, they proposed to build a new training center to accept more youths and prevent them from using substances.

After analyzing the responses from teachers, we designed surveys for *students* currently enrolled in the training centers. A total of 18 responses were collected from the two training centers at Totonga and Elikiah. All respondents are above 18 years old, with nine individuals (50%) falling within the age range of 18 to 24, five individuals (28%) aged over 24, and the remaining respondents (22%) not providing detailed information on their age. (Fig. 4) Regarding the transportation questions, six respondents (33%) stated that they could reach the training center within an hour, while nine respondents (50%) reported spending more than 60 min commuting to the training center (three respondents did not answer this question). (Fig. 5) Most students indicated that they walk to the training center, although some mentioned occasionally biking. All students reported that they spend more than 8 h at the training center each day, attending five days a week, which is consistent with the information provided by the teachers. About one third of the students (28%) come to the training center through outreach by youth leaders, three individuals (17%) heard about it from radio and two students (11%) learned about it from living forces meetings. (Fig. 6)

We also sent surveys to the *youth leaders*, which is another group of key individuals providing support to the training centers. Youth leaders play a crucial role in locally engaging with young individuals and introducing them to the training centers. We received over 35 responses from youth leaders, who come from four main chiefdoms within the Aru Territory, Ituri Province: Kaliko-Omi (20%), Zaki (31%), Aluru (31%), and Commune D’ Aru (18%). Commune D’ Aru is the chiefdom of the territory’s administrative capital, Aru Town. (Fig. 7). Nevertheless, the youth leaders are spread throughout the entire Aru Territory, recruiting youth from various locations. Many of these leaders are associated with the Church, communicating in Lingala which is the local language used in Aru. Of the respondents, 32 individuals (91%) are male, with ages ranging from 18 to 40 years. They use various outreach methods, such as WhatsApp, radio, wall advertisements, cell phones, or door-to-door visits, to recruit youth for the training centers. Typically, they reach out to the youth once a week and record their interactions on paper. Each person who qualifies might be contacted two to three times, and there is considerable interest expressed in attending classes in a training center. As mentioned by the youth leaders, not every interested individual can secure a spot at the training centers due to limitations in both teaching staff and physical space.

## Discussion

This study explored how community training programs in Aru, DRC, contribute to community development and social engagement that may serve as protective factors against youth substance use. However, the preventive effect of these centers was not measured and remains a hypothesis for future research. To better present our findings, we developed a comprehensive process map that visually illustrates the roles and interactions of key stakeholders, including local churches, community workers, and non-governmental organizations (NGOs). This process-mapping approach clarifies the structure of existing community efforts. Adams (Adams, 2020) also utilized a process mapping approach similar to our study to identify community efforts in preventing substance use [17]. The process map in Adams’ study (Adams, 2020) served as a valuable tool for illustrating how key stakeholders are working together within the system, helping them recognize their roles in addressing opioid use and recovery [17]. Their findings were primarily derived from interviews with various stakeholders. In contrast, our study used a mixed-methods approach, integrating both qualitative and quantitative data to enhance the validity of our findings and provide a more comprehensive understanding of community efforts in substance use prevention in Aru, DRC.

Although the study was conceptualized to examine substance use among youth aged 15–24 years, as defined by the World Health Organization (WHO), all our participants were aged 18 years and older. This demographic pattern was not an intentional inclusion criterion but rather an unexpected finding resulting from the local enrollment data of the training centers. The training centers primarily serve young adults who have left the military and face limited job opportunities, which likely accounts for the absence of minors in the study population. Despite this age distribution, maintaining the term “youth” still remains appropriate within the community-engaged context of the Aru Diocese. The term “youth” is consistently used by local authorities and organizations to describe all individuals enrolled in training centers or rehabilitation programs. Retaining this terminology reflects the local sociocultural understanding of “youth” while acknowledging that the study findings pertain specifically to young adults aged 18 and above. Future studies may benefit from examining differences between adolescents and young adults within this population to better understand age-related variations in substance use behaviors and intervention needs.

Researchers are increasingly recognizing the importance of including input from non-English-speaking participants in the research process [24]. However, there is limited literature on effectively engaging these communities in resource-limited settings, particularly regarding communication. Squires and the team [25] provided the best practices for conducting research with individuals facing language barriers, emphasizing the need for methodological rigor [25]. Our study aligns with these recommendations by implementing structured approaches to cross-language research. To capture diverse perspectives, we identified four key groups in the community effort: youths, the leadership team, teachers, and youth leaders. We then developed tailored surveys for each group. This approach ensures that all voices were represented and contributed meaningfully to the evidence informing policies and public services. By applying strategies consistent with Squires and the team [25], we aimed to minimize misinterpretation and enhance data accuracy [25]. Our findings contribute to the growing body of cross-language research and provide a foundation for future studies to refine methodologies that promote more inclusive and representative research practices.

During the research process, our project encountered several difficulties regarding language barriers. We want to list these difficulties here for future research that might apply community-engaged research with non-English-speaking participants in extremely resource-limited communities. Firstly, analyzing responses to open-ended surveys is time-consuming and challenging, as it requires more effort in reviewing, interpreting, codifying, and categorizing the results. Additionally, we needed to use paper-based surveys since the local situation is lack of electricity. Many handwritten responses to our open-ended questions were difficult to interpret, resulting in the omission of important information in the initial attempts. Secondly, significant language barriers exist between the two countries involved in the research process. French is the official language in the DRC, but people in the Aru region speak several local languages like Swahili, Lingala, Ndo, etc. Despite using a translator or electronic translation tools, the research team encountered challenges in seamlessly translating our questions from English into various languages and translating the responses back into English. The collected responses sometimes deviated from the intended questions due to inaccurate translations and misunderstandings. Lastly, reading literacy and writing literacy levels vary among each key individual group and across age group.

We utilized three specific strategies to communicate effectively with communities that face resource limitations and language barriers. The first strategy we used was to arrange the survey order based on the English proficiency and literacy levels of the interviewees. In this study, we collaborated with English-speaking community workers to gain insights into the local situation and drafted an initial process map. After collecting basic information and creating the initial map, we were engaged with local training center teachers who have strong communication skills but do not speak English. Finally, we adjusted our survey approach to include youths with the lowest literacy level. They are an important stakeholder in the whole process but the most challenging participant group when it comes to designing surveys for them. The second strategy we used was to select a format to ensure that research materials are accessible to all participants, including those with low literacy levels. In our study, we decided to use a closed-ended survey format for the non-English-speaking community. To make the survey understandable, we provided a more detailed explanation for each question and used simple language and words when designing the questions and answer choices. The third strategy we implemented was to hire bilingual staff members or translators to ensure accurate communication. This is also a key recommendation provided by the literature [25]. This strategy improves communication quality on both ends. These strategies have been successful in enabling us to gather more information from various key individuals. Implementing them helped us finalize our initial process map.

Although alternative participatory methods such as oral storytelling, group discussions, or participatory mapping might have provided richer qualitative insights, these approaches would have required significant additional time and translation resources for implementation and analysis. Because local community partners assisted with translation on a volunteer basis, written surveys were selected as the most feasible approach for this study. This decision was also informed by our community partners, who advised that written surveys would be the most practical and inclusive method for reaching youth leaders, teachers, and students across multiple training centers and locations. While this approach may have limited the narrative depth of responses, it enabled broad participation across geographically dispersed sites and minimized burden on community partners. In addition to this limitation, there are several other limitations in our study that should be addressed. Firstly, we had a small sample size as we could only collect data from two training centers in the Aru area. Specifically, we focused on teachers and students in the training centers close to the Aru City, which means that the results found may not apply to other training centers. We selected these two training centers because they were located near the urban area and were closer to the community partners. Future studies should aim to include a larger and more geographically diverse sample to enhance generalizability and capture the perspectives of students and teachers in different settings across the Aru Territory. Secondly, we did not collect detailed personal information about the participants. To ensure that participants felt comfortable sharing their experiences, we only asked for their first and last name initials on the survey page. However, some participants did not even provide an initial on the survey page, which made it difficult for us to analyze the survey results. Future research could adopt alternative strategies for balancing anonymity with data richness, such as assigning coded identifiers to better understand subgroup variations without compromising participant privacy. Lastly, it is possible that some of the options we provided in the closed-ended survey were not accurate and may have influenced the results. We tried to minimize potential biases and misunderstandings by providing a detailed introduction on the first page of the survey, it explained the purpose of the survey and gave examples of how to answer this survey. We also gave participants an additional option labeled “Other” on every question to provide their own answers in written format. However, potential biases and misunderstandings could still exist due to the large language barriers. Future studies could improve data accuracy by pretesting survey instruments with a small pilot group to refine question wording and response options based on participant feedback.

## Conclusion

This study developed a systematic understanding of how community training programs operate and engage with youth in Aru, Democratic Republic of Congo. The findings highlight these centers as important locally recognized assets that may contribute to addressing youth substance use challenges. However, their preventive impact has not yet been formally evaluated and should be explored in future studies. Future research should test this hypothesis using longitudinal or observational designs to better understand the relationship between training center participation and substance use outcomes. Transportation barriers emerged as a key challenge, with both teachers and students reporting that many youths wish to attend the training centers but are limited by distance and the insufficient capacity of existing facilities. These findings underscore the need to establish an additional training center in a more accessible location to better serve surrounding communities. Future research should build on this study by assessing the preventive effects of these centers, exploring strategies to expand access, and evaluating how logistical improvements, such as transportation support or decentralized program locations, might enhance youth engagement. By addressing these structural barriers, efforts to prevent youth substance use in Aru, DRC can be strengthened and sustained.

## Supplementary Information

Supplementary Material 1.

Supplementary Material 2.

## Data Availability

All data collected for this study were de-identified to protect participant confidentiality. The de-identification process included: a) Assigning each study subject a unique “study subject ID” to replace personal identifiers in the research data.b) Maintaining a separate, password-protected code key—stored securely in a Box folder—that links participant names with their assigned IDs. This code key is accessible only to designated research team members.De-identified data is stored and analyzed in a secure Box folder accessible only to the research team. The datasets generated and analyzed during the current study are available from the corresponding author upon reasonable request.
