# Supplementary material for: Towards an understanding of community-engaged efforts in addressing the youth substance use in the Democratic Republic of the Congo
Source: BMC Public Health. 2025 Dec 19;26:278. doi: 10.1186/s12889-025-25809-z (PMC12829273; doi:10.1186/s12889-025-25809-z)
Supplement: Supplementary file 1 — Supplementary Material 1. [file 12889_2025_25809_MOESM1_ESM.docx]

Appendix A: Open-ended Survey for Religious Leaders

**Contextual Information Gathering from Religious Leaders in Aru Diocese, DRC**

**Background:**

Youth substance abuse is both a result and a cause of complex social problems such as poverty, school dropout, lack of educational and economic opportunity, and lack of awareness about the consequences of substance abuse. This project intends to create a community-based support network between Purdue University and members from World Concern, local churches, religious leaders, training centers, community workers, and Salama University to provide (1) early interventions and basic education/job training to at-risk youths and (2) detoxification and recovery services to youths suffering from substance abuse. This project will take several stages, with the current stage focusing on understanding the demand of at-risk youths and available resources for youth development services.

**Purpose of the Questionnaire:**

The primary task for the Purdue University team at this stage is to understand the current situation by identifying (i) important individuals and groups in the process of providing early intervention and basic training to at-risk youths and (ii) how different individuals and groups interact with each other. This will help set the foundation for making recommendations to improve the current practice.

In the appendix, we propose a “conjectured” process map of addressing youth substance abuse in Aru Diocese based on our collected preliminary information. This questionnaire primarily serves the purpose of understanding the role of religious leaders in this process and getting their perspective on how community workers/teachers interact with youth.

This questionnaire is in the form of open-ended questions. Additional descriptions are provided for each question to provide contextual information. However, some of these descriptions may be inaccurate due to the limited understanding from the Purdue University team. Please provide responses to all questions below to help ensure our descriptions are accurate as possible.

**Please answer the following questions to the best of your ability. If you come across a question that you do not know or are unsure, respond with “I do not know” or “I am unsure.”**

1. We would like to understand the individuals and groups involved in the process of addressing youth substance abuse.

Based on the information we obtained, we identified four types of individuals and groups by their roles:

- Youths affected by substance: youths at risk and youths actively affected by substance, those who the community-based support network wants to help.
- Religious leaders: organizers and coordinators.
- Community workers such as youth leaders and staff for World Concern: the core of the process, executors linking the youths, religious leaders, and teachers in the training centers together.
- Teachers in the training centers: conducting the teaching.

Does this categorization of four types of individuals and groups sound about right to you? If not, please help us identify the individuals and groups and describe their roles in the process. For their roles, please elaborate on how they contribute to addressing the issue of youth substance abuse.

2. From your perspective, what are the biggest challenges you think in the current process of helping youths and preventing them from using substance?

Here are a few examples for the challenges:

- There are not enough training centers.
- There are not efficient ways to attract youths to participate in the training centers or to keep them.
- Military group activities may affect the daily operation of the training centers.

3. The following questions are used to collect information on the background of youth substance abuse in Aru Diocese:

1. What are the factors do you think that have caused youth to use substance in Aru Diocese?
2. Youth population information:

- Based on the information we collected online, we estimate the total population of the Aru Diocese is around 980,000. Among them, around 193,000 are youth between 15-24 years old. Do these estimates sound about right to you? If not, please help us correct it.
- How many youths in Aru Diocese do you think may be at risk and need help? If you have not done any census before, could you help us roughly estimate the percentage of youth that you believe may be at risk? For example, out of our estimated 193,000 youth population, what percentage of youth do you believe are at risk of using substance?

4. The following questions are for us to understand what roles religious leaders play in addressing youth substance abuse. The questions are open-ended with some examples (may not be accurate) to help explain the questions. Please elaborate based on your primary duties/responsibilities.

Regarding youth substance abuse:

1. What are the primary activities you and your organization do? What does your workflow or process involve?
2. Do you directly interact with youths at-risk?

- If you directly interact with youths at-risk, what kind of support do you provide to them? Examples include providing religious training to help them build faith in life, spirit-lifting speech, etc.

1. Whom do you interact with frequently in your primary activities?
2. How often do you meet with each of the individuals and groups identified in questions III)?
3. What are the topics of your meetings?
4. What kind of support do you provide to the individuals and groups you interact with?

Please give us a few examples; this may help us better understand the process. For example, leadership training (training religious leaders), support on resources (funding), etc.?

1. What kind of feedback do you receive from the individuals and groups working with you?

Please give us a few examples; this may help us better understand the process.

- What is the format of feedback, oral presentation or written report?
- What is included in the feedback?

5. The following questions are to help us understand the roles of training centers (teachers) and youth leaders. Answer from your perspective and understanding.

1. About training centers:

- How many training centers are there in the Aru Diocese?
- What are the main subjects taught in each training center, e.g., sewing, wood carpentry, etc.?

1. About teachers in the training centers:

- Who are the teachers in the training centers?

1. Are they residents in the community?
2. Are they affiliated with primary schools or with local churches?

- Do you have a rough estimate on how many teachers a training center usually has?
- How do training centers recruit teachers?

1. About youth leaders:

- Who are the youth leaders?
- Are youth leaders mainly affiliated with the churches?
- Do you have a rough estimate on how many youth leaders a village or region usually has?

1. The roles of youth leaders:

- What roles do youth leaders play? For example, do they mainly interact with youths and connect them to join the training centers?
- How are youth leaders recruited?
- What is the literacy, numeracy, and working language of youth leaders?

1. Besides youth leaders, who are other important community workers that we have not mentioned here? Please give some examples.

6. Please comment on the process map we have created (see appendix) and give us some suggestions to more accurately describe the process of addressing youth substance abuse via the efforts of all individuals and groups.

Appendix B: Open-ended Survey for Teachers

**Information Collection for Teachers in Training Centers**

Background

Youth substance abuse is both a result and a cause of complex social problems such as poverty, school dropout, lack of educational and economic opportunities. Supported by the World Concern, Purdue University, Salama University, this project intends to create a community-based support network in Aru Diocese, consist of local churches, community leaders, training centers, and community workers to provide.

(1) Early interventions and basic education/job training for at-risk youths;

(2) Detoxification and recovery services to youths suffering from substance abuse.

This project will take several stages, with the current stage focusing on understanding the demand of at-risk youths and available resources for youth development services.

Purpose of the questionnaire

Teachers in the training centers are critical in the process of providing youth professional with skills and conflict-solving techniques. Through meetings with Aru team and the first-round of survey, we have identified that (i) the lack of teachers and (ii) the lack of resources in coaching and retaining these teachers are major barriers in attracting more at-risk youth to the training centers and helping them success after the program. The primary purpose of this questionnaire is to facilitate our understanding of the operations of the training centers and help us collect more information about the challenges in attracting and retaining teachers in the training center. The end goal is to understand where more resources should be placed to attract, coach, and retain teachers in the training centers.

This questionnaire is in the form of open-ended questions. Additional descriptions are provided for each question to provide contextual information. However, some of these descriptions may be inaccurate due to a limited understanding of the Purdue University team. Please provide responses to all questions below to help ensure that our descriptions are accurate as possible.

Please answer the following questions to your best. If you come across a question that you do not know or are unsure about, respond with “I do not know” or “I am not sure.

1. Basic information.

- What is your name?
- Are you a male or female?
- What is your age?
- Have you always lived in Aru? If not, where did you live before coming to the Aru? For example, Kananga or other parts in Congo?
- Are you affiliated with the local church, or primary schools, or other organizations? If you are from other organizations, could you please tell us which organization you are affiliated with?
- What language(s) do you speak? For example, French, Kikongo (Kituba), English?
- What subjects do you teach? For example, carpentry, dressmaking, adult literacy?
- Before becoming a teacher in this training center, did you have another job? If yes, what job did you have and where was that job?
- Who recruited you to become a teacher in this training center? For example, the local church, World Concern, or the Five Talents?

1. About workflow.

- What are your daily activities?

Describe one of your typical days. What do you do in the morning and in the afternoon? For example, in the United States, a teacher gives students lectures, help students practice the skills. Do you do similar things?

- How many students are you teaching right now?
- Normally how long do students stay in the training center? 3 months? 6 months? One year?
- What are the major difficulties you have in your daily work? For example, when you deliver your teaching, what things make the teaching difficult? Example could include lack of means of transportation for students, teachers, researchers, internet connection problem, lack of electrical energy, lack of documents necessary for training, disruption from militant groups.
- What additional resources will help you work better? For example, more teaching materials, more supporting staff, or monetary support.

1. Interaction and collaboration with other groups.

- Where did you get trained to become a teacher in this training center?
- Do you interact with religious leaders in local churches?
- Do you interact with youth leaders and community workers?

1. About youth using substance.

- What factors do you think that drive youth to use substance in Aru? For example, not having a job, dropping out from schools.
- How can the training center reach out to more youth who are at-risk of using substance? How can the training center recruit more youth to become students?
- How could the training center be improved?

1. Open question.

- Tell us about your experiences of being a teacher in this training center. For example, how did this experience impact your life?

Appendix C: Semi-Structured Interview Guide

**Semi-Structured Interview Guide**

**Introduction:** To start off, I will go ahead and introduce myself and describe the process of this interview. My name is XXX, and I am a researcher at Purdue University in the United States. Thank you for taking the time to meet today! The interview should take around 30-60 minutes, is that okay?

We are interested in understanding more about your teaching style, difficulties that may exist in teaching students, and ideas on how we can improve these difficulties. I will be recording the audio of today’s meeting, but as a reminder, you can ask me to skip any questions, pause, or stop the interview at any time. [Also, please do not use names of student or people you work with or share information that could reveal who someone is during the interview. We will remove anything that could identify someone and summarize the interview results for our community partners and for publishing purposes.

I may write down some things as you talk, I am still listening but want to make sure I am keeping track of important information you share.

Before I start the recording, I want to ask you a few demographic questions. If at any time you feel uncomfortable with a question, you can say that you “prefer not to answer.”

1. **Collect demographics** (we can collect this information before the interview)
2. Age
3. Gender
4. Teaching role
5. Number of students
6. Language
7. Local church affiliation

I am going to start the recording now.

Semi-structured interview will include questions such as

1. **Relationships:**

**Teacher training**

Where did you receive your teaching training? How did you find [school name]?

How many subjects are taught at the teacher training center?

Are you familiar with using technology (such as computer) in your teaching?

Do you use WhatsApp? Do you use Google or other social media apps?

**Student training**

What subject(s) do you teach to students?

How do adolescents find the training center and become students?

Is there a fee associated with training?

Can students choose the training area they participate in?

How many hours do you train students in [subject] a day?

How long are your classes?

What are your daily activities while teaching at the training center?

**Focus the remaining time on the sections below:**

*[Interviewer: In the next set of questions, we will discuss issues that make training the youth difficult.]*

**3.Current barriers:**

In your opinion, what prevents youth/students from attending training?

What kind of resources do you use in your training? Number of supplies?

How does the training center recruit students? Can students join the training at any point during the year?

Do you use electricity every day?

What resources would allow you to teach more students?

How do students find a job once they complete the training?

How do you think student attendance/completion can be improved?

How do you document student progress? Do you think a technology solution would help this documentation?

*[Interviewer: In the next set of questions, we will discuss possible interventions that could be made to support the training center.]*

**4.Interventions:**

Do you track student attendance and progress through the training program? If yes, how do you track the progress for each student?

How do you track your teaching workload and responsibilities? Do you have a phone or computer? If yes, does your phone have access the internet? What other electronic devices do you use during teaching (i.e. computer)?

Appendix D: Close-ended Survey for teachers

**Information Collection for Teachers in Training Centers**

Background

Youth substance abuse is both a result and a cause of complex social problems such as poverty, school dropout, lack of educational and economic opportunities. Supported by the World Concern, Purdue University, Salama University, this project intends to create a community-based support network in Aru Diocese, consist of local churches, community leaders, training centers, and community workers to provide

(1) Early interventions and basic education/job training for at-risk youths;

(2) Detoxification and recovery services to youths suffering from substance abuse.

This project will take several stages, with the current stage focusing on understanding the demand of at-risk youths and available resources for youth development services.

Purpose of the questionnaire

Teachers in the training centers are critical in the process of providing youth professionals with skills and conflict-solving techniques. Through meetings with Aru team and the first-round of survey, we have identified that (i) the lack of teachers and (ii) the lack of resources in coaching and retaining these teachers are major barriers in attracting more at-risk youth to the training centers and helping them success after the program. The primary purpose of this questionnaire is to facilitate our understanding of the operations of the training centers and help us collect more information about the challenges in attracting and retaining teachers in the training center. The end goal is to understand where more resources should be placed to attract, coach, and retain teachers in the training centers.

Please answer the following questions to your best.

1. Are you a male or female?

- Male
- Female

1. What is your age?

- Less than age 20
- 20-39
- 40-59
- 60-79
- Older than 80

1. What transportation do you take to come to work?

- Walk
- Bicycle
- Car
- Bus

1. Which organization are you affiliated with?

- Anglican church of Congo
- Others

1. What subjects do you teach?

- Carpentry
- Dessin coupe
- textile technology

1. How many days per week do you come to the training center?

- Less than 1 day
- 1 to 2 days
- 3 to 4 days
- 5 to 6 days
- 7 days
- Others

1. What time do classes usually start?

- Morning
- Afternoon
- Evening

1. How long are your classes?

- Less than 2 hours
- 2 hours
- More than 2 hours

1. How many classes do you teach per day?

- 1 class
- 2 classes
- 3 Classes
- More than 3 classes

1. How many hours do you work per day?

- Less than 8 hours
- 8 hours
- More than 8 hours

1. How many students are you teaching right now?

- Less than 10
- 10 to 20
- 21 to 30
- 31 to 40
- More than 40

1. How long do students stay in the training center?

- Less than 6 months
- 6 months
- Between 6 to 10 months
- 10 months
- More than 10 months

1. What do you think is the major issue that prevents students from learning?

- Transportation
- Cost
- lack of teaching materials
- electricity

1. How does the students come to the training center?

- Bus
- Bike
- Walk
- Others

1. Can students choose what classes they want to take in the training center?

- Yes
- No

1. Have you received any training before you become a teacher in this training center?

- Yes
- No

1. What factors do you think leading to the teenagers to take drugs in Aru?

- Not having a job
- dropping out of school
- Others (please provide comments:___________)

1. Open question:

How could the training center be improved? What are the major issues the training center faces right now?

Appendix E: Close-ended Survey for Students

**Information Collection for Students in Training Centers**

**Purpose of the questionnaire**

Through meetings with the Aru team and doing the first and second-round surveys with religious leaders and teachers, we have identified some difficulties in attracting more at-risk youth to the training centers and helping them success after the program. However, we would like to understand the current situation from the student’s perspective and the difficulties for them to complete the training program.

The questionnaire is designed to collect individual student information and it can be done in either of the following option:

1. Student finish the questionnaire themselves.

2. Someone assist the students to complete this survey. (For example, give this survey to a teacher in the training center and ask this teacher to talk to the student individually and ask the following questions. The teacher should mark down student’s respond by selecting one answer from the multiple choices.)

However, some of these descriptions may be inaccurate due to the limited understanding of the Purdue University team. Please provide responses to all questions below and provide additional information if none of the choices provided fit the situation to help ensure that our descriptions are accurate as possible.

Here is an example of how these questions could be answered. (all the questions designed from the student’s perspective. Therefore, “you” refer to the student who takes the survey.)

Example question:

| What classes do you take in the training center? (If more than one, choose all that apply) | - Dessin coupe - General seams - Textile technology - Carpentry - Others (______________) |
| --- | --- |

| **Basic information** | |
| --- | --- |
| Are you a male or female? | - Male - Female |
| What age range do you fit in? | - Younger than age 14 - Age 14-16 - Age 16-18 - Age 18-20 - Age 20-22 - Age 22-24 - Older than age 24 |
| What did you do before coming to the training center? | - Went to school - Had other jobs - Unemployment at home - Others (________________) |
| **Transportation** | |
| How much time do you take to come to the training center from where you lived? | - Less than 30 minutes - 30-60 minutes - More than 60 minutes |
| How do you come to the training center? | - Bus - Walk to school - Bicycle - Others (please be specific if possible) |
| **About the training center** | |
| How do you know about this training center? | - From meetings of the living forces - Religious leaders reach out - Youth leaders reach out - Community workers reach out - From University Salama d' Aru - Others (please be specific if possible)   ____________________________ |
| What classes do you take in the training center? (If more than one, choose all that apply) | - Dessin coupe - General seams - Textile technology - Carpentry - Others (___________________________________) |
| How useful do you feel the classes you take will help you find a job? | - Very useful - Somehow useful - Not so useful - Not useful at all |
| How long will you stay in the training center? | - Less than 6 months - 6 months - 10 months - Longer than 10 months |
| How long do you spend in the training center per day? | - Less than 2 hours - 2-4 hours - 4-6 hours - 6-8 hours - More than 8 hours |
| How often do you come to the training center per week? | - Once per month - Two to three times per month - Once per week - Two to three times per week - Four to five times per week - More than five time per week |
| What do you wish to get from the training center? | - Get a job - Learn a useful skill - Start a business - Others (be specific)   _________________________________ |
| How can the training centers be improved? What are their major difficulties right now? | - Transportation to the training center - Cost (or funding) - Electricity (or internet connection) - Teaching materials - Need more teachers - Need more students to participate - Increase Building size |
| **About this Survey** | |
| Since this survey is designed in English and then got translated to Lingala, there might be some translation errors. Overall, how do you feel about this survey? Is this survey easy to understand? | - Very easy to understand - There are some translation errors, but we can still understand the questions and answers - There are some difficulties when understand this survey - The questions and answers are hard to understand |
| Open questions (optional)  What other things do you think can the training center do to help you learn better? | |

Appendix F: Close-ended Survey for Students

**Information Collection for Youth Leaders**

**Purpose of the questionnaire**

Through meetings with the Aru team and doing the first and second-round surveys with religious leaders, teachers and students, we have identified (i) the lack of resources in the training centers to accept more students, and (ii) the lack of transportations are major barriers in retaining more at-risk youth at the training centers and helping them success after the program. To understand the feasibility of providing transportation and estimating the demand of students, we want to know more from youth leader’s perspective.

We believe that youth leader plays an important role in outreaching and connecting local youths to the training centers. Therefore, we designed this survey to better learn from the youth leader’s perspective and get to know more about their workflow.

**Youth leaders**: your opinions are crucial for our researchers to understand how youths are attracted to training centers and how could we expand the resources to better help them.

Please provide responses to all questions below. Provide additional information if none of the choices provided fit the situation. Here is an example of how these questions could be answered. All the questions designed from the youth leader’s perspective. Therefore, “you” refer to the youth leader who takes the survey.

| Are you a male or female? | - Male - Female |
| --- | --- |

Since this survey is five pages long and we want to collect the complete response from one person, please put the first letter of your first name (given name or forename) and last name (Surname or family name) on top of this questionnaire so that we could identify the same response from one person. For example: if your name is Gloria Ngoy, please write down GN on top of every page of your survey.

| **Basic information** | |
| --- | --- |
| Are you a male or female? | - - - Male     - Female |
| What age range are you in? | - Younger than 18 - Age 18-24 - Age 25-30 - Age 31-35 - Age 36-40 - Older than 40 |
| What organizations are you affiliated with? | - Local Churches - Government - Woman Association - Local Peace Committees - Self-employed - Others (________________________) |
| **About Roles and Workflow** | |
| What kind of support do you offer to the youth? If you provide more than one service, please select all that apply to you. | - Recruit the youth to the training center - Provide peer support - Organize educational meetings - Work with local church - Work with community workers - Work with medical professionals - Work with local farmers - Others (____________________________) |
| What language do you mainly use to communicate with the youths from the villages? | - French - Lingala - Other (________________________) |
| Do you use a translator to facilitate communication with youths speaking different languages? | - - - - Yes       - No |
| What methods do you use to attract youths to join the program? If you used more than one method, please select all methods you used on the list. | - Whatsapp or other social media - Radio - Poster or flyer, advertisement - Walk door to door - Cellphone or phone calls - Others (_______________________) |
| How often do you reach out to the youth on average? | - Once every two weeks - Once a week - Twice a week - Three to four times per week - Five times a week or more - Others (_______________________) |
| How many youths do you reach out per month on average? | - Less than 5 - 6 to 10 - 11 to 20 - 21 to 30 - More than 30 |
| How many times do you usually reach out to one youth before he/she becomes interested in attending the training center? | - - - Only once     - Two to three times     - More than three times |
| What are the local youths’ overall attitude toward attending training centers? | - Most of them interested in attending. - About half of them interested. - Less than half of them want to go to the training center. - They are not interested in going to the training center at all. |
| For youth who are interested in attending the training center, can they usually be accepted? | - Yes - No |
| If not, how many of youth you reached out in the last year were declined from attending the training center? | - Less than 10 students. - About 10 to 20 students - About 21 to 30 students. - About 31 to 40 students. - More than 40 students. |
| **Technology** | |
| What method do you use to record the number of youths you reach out per day? | - Record it on paper - Use computer - Use cellphone - Others (_____________________) |
| Are you having access to the internet every day? | - Yes, always have internet access - Sometimes but not always, the connection is not stable. - Not have access at all. |
| What kind of device do you use to get access to the internet? | - By cellphones - By computers - Others |
